# Gender Differences in Cerebral Regional Homogeneity of Adult Healthy Volunteers: A Resting-State fMRI Study

**DOI:** 10.1155/2015/183074

**Published:** 2015-01-01

**Authors:** Chunsheng Xu, Chuanfu Li, Hongli Wu, Yuanyuan Wu, Sheng Hu, Yifang Zhu, Wei Zhang, Linying Wang, Senhua Zhu, Junping Liu, Qingping Zhang, Jun Yang, Xiaochu Zhang

**Affiliations:** ^1^Medical Imaging Center, The First Affiliated Hospital of Anhui University of Chinese Medicine, Anhui 230031, China; ^2^Laboratory of Digital Medical Imaging, The First Affiliated Hospital of Anhui University of Chinese Medicine, Anhui 230031, China; ^3^Institute of Computer Application of Chinese Medicine, Anhui Academy of Chinese Medicine, Anhui 230038, China; ^4^Graduate School, Anhui University of Chinese Medicine, Anhui 230038, China; ^5^Department of Electronic Science and Technology, University of Science & Technology of China, Anhui 230027, China; ^6^Institute of Biophysics, Chinese Academy of Sciences, Beijing 100101, China; ^7^School of Acupuncture & Osteology, Anhui University of Chinese Medicine, Anhui 230038, China; ^8^Department of Acupuncture and Moxibustion, The First Affiliated Hospital of Anhui University of Chinese Medicine, Anhui 230031, China; ^9^CAS Key Laboratory of Brain Function & Disease and School of Life Sciences, University of Science & Technology of China, Anhui 230027, China; ^10^Center of Medical Physics and Technology, Hefei Institutes of Physical Science, CAS, Anhui 230031, China

## Abstract

*Objective.* We sought to use the regional homogeneity (ReHo) approach as an index in the resting-state functional MRI to investigate the gender differences of spontaneous brain activity within cerebral cortex and resting-state networks (RSNs) in young adult healthy volunteers. *Methods.* One hundred and twelve healthy volunteers (56 males, 56 females) participated in the resting-state fMRI scan. The ReHo mappings in the cerebral cortex and twelve RSNs of the male and female groups were compared. *Results.* We found statistically significant gender differences in the primary visual network (PVN) (*P* < 0.004, with Bonferroni correction) and left attention network (LAtN), default mode network (DMN), sensorimotor network (SMN), executive network (EN), and dorsal medial prefrontal network (DMPFC) as well (*P* < 0.05, uncorrected). The male group showed higher ReHo in the left precuneus, while the female group showed higher ReHo in the right middle cingulate gyrus, fusiform gyrus, left inferior parietal lobule, precentral gyrus, supramarginal gyrus, and postcentral gyrus. *Conclusions.* Our results suggested that men and women had regional specific differences during the resting-state. The findings may improve our understanding of the gender differences in behavior and cognition from the perspective of resting-state brain function.

## 1. Introduction

A large number of postmortem and imaging investigations on gender differences were dedicated to the human brain [1–7]. Previous structural neuroimaging studies showed that the brain size is larger in men than in women and revealed that the anatomical gender differences of the brain in the cortex are region-specific [8, 9]. Women usually have larger relative volumes in frontal and medial paralimbic cortices, while men have larger frontomedial, amygdala, and hypothalamus relative to cerebrum size [9, 10]. Apart from these anatomical differences, gender differences also exist in many behavioral and cognitive domains. Men generally perform better in visual and spatial processing as well as mathematics [11–13], whereas women tend to outperform men in verbal skills and memory [14, 15], facial emotion recognition [16], fine motor skills [4], and emotion processing [4, 17].

Not only is magnetic resonance imaging a significant imaging modality in clinical diagnosis and treatment planning, but it also provides detailed knowledge of physiological and pathological brain functions for medical research [18]. By taking the advantages of fMRI technique, many studies have reported gender differences in the functions of the brain, such as the sex-related hemispheric lateralization in language processing [19] and emotional memory [17], suggesting that male and female brains might have some different neural mechanisms to conduct certain tasks [20]. However, compared with these task state studies, there are still few studies on the difference in the resting-state of the brain. The resting-state function reflects the neuronal baseline activity of the brain when the subject is not performing an explicit task, representing the state of the human brain in the absence of goal-directed neuronal action and external input. Biswal et al. [21] investigated sex effects using amplitude of low-frequency fluctuation (ALFF) in a large-scale resting-state fMRI cohort (*n* = 1414, across 35 imaging centers) and suggested that sex emerged as a significant determinant. Wang et al. [13] employed a support vector machine-based multivariate pattern analysis (MVPA) approach and found men showed higher regional homogeneity (ReHo) in the right hemisphere and women tended to show greater ReHo in the left.

The ReHo is defined as the regional synchrony of spontaneous fMRI signals and can be used to map the resting-state brain function [22]. To investigate the gender difference in the brain functions, the first question is whether there is any gender difference in ReHo within the resting-state networks (RSNs). There are about twelve RSNs in the brain as previously reported, such as the default mode network (DMN), the sensorimotor network (SMN), the visual network (VN), the auditory network (AN), the salience network (SN), and the attention and executive function networks [23, 24]. The second question will be what specific brain areas are. To address these two questions, we analyzed the gender difference of ReHo in twelve RSNs and whole cerebral cortex of adult healthy volunteers. We hypothesized that men and women might have different ReHo in some of the RSNs and cerebral cortices, which might be related to the gender differences in cognitive domains as previously reported [1–7].

## 2. Materials and Methods

### 2.1. Subjects

One hundred and twelve healthy volunteers (56 males, 56 females) were recruited to take part in this experiment. All the procedures were fully explained to the participants and informed consents were obtained from all subjects before they took part in the experiment. All volunteers were the college students in the university, right-handed with no history of mental or neurological disease, with no history of psychiatric and neurological disorders or drug use, and with no obvious abnormality in brain structure. This study was approved by the Ethics Board at the First Affiliated Hospital of Anhui University of Chinese Medicine.

### 2.2. Data Acquisition

Before the experiment, the participants were requested to change clothes, rest, and then enter into the scanning room after the whole body had been relaxed. The subjects were told to close their eyes and their ears were stuffed with cotton balls during scanning. The lights in the scanning room were turned off to reduce visual stimulation. During the entire scanning process, the subjects were asked to avoid psychological activity as far as possible.

All fMRI experiments were completed at the MR room of the Medical Imaging Center, the First Affiliated Hospital of Anhui University of Chinese Medicine. The Siemens Symphony 1.5T MRI whole body scanner (Siemens Medical Systems, Germany) and standard head coil were used. A total of 4 sequences were scanned, which were as follows. (1) The first sequence was pilot images. (2) T2-weighted images: this sequence lasted for 1 minute 30 seconds. The goal of this sequence was to find whether or not there was any obvious structural abnormality of the brain. (3) Resting-state fMRI data acquisition: this sequence lasted for 10 minutes. Take the axial position parallel to the AC-PC line, with 36 slices that covered the whole brain. EPI-BOLD sequences were used, with TR/TE/FA of 3000 ms/30 ms/90°, FOV of 192 mm × 192 mm, and matrix of 64 × 64. (4) T1-weighted 3D anatomical images: this sequence lasted for 8 minutes 59 seconds. Sagittal position was taken and 176 slices were scanned which covered the whole brain. Spoiled gradient echo sequence was used, with TR/TE/FA of 2100 mm/3.93 mm/13°, FOV of 250 mm × 250 mm, slice thickness/spacing of 1.0 mm/0.5 mm, and matrix of 256 × 256. It took about 21 minutes to complete all the data acquisition.

### 2.3. Data Preprocessing

All preprocessing procedures were performed using the AFNI software (http://afni.nimh.nih.gov/) in the Laboratory of Digital Medical Imaging, the First Affiliated Hospital of Anhui University of Chinese Medicine. The first 4 volumes of the functional images were discarded for the signal equilibrium and participants' adaptation to the scanning circumstance. After excluding the first 4 volumes, all fMRI volumes were slice corrected and then realigned to the first volume. Data was included if the subject's head movement during fMRI scanning was less than 2 mm translation and less than 2° angular rotation in any axis. Six motion parameters, linear drift, and the mean time series of all voxels within the white matter and the cerebrospinal fluid were removed from the data by linear regression to reduce the effects of confounding factors. After that, a temporal filter (0.01–0.08 Hz) was applied to reduce the effect of low-frequency drift and high-frequency physiological respiratory and cardiac noise signals.

### 2.4. ReHo Analysis

The ReHo analysis was performed in each subject with the program of 3dReHo in the AFNI software. Kendall's coefficient of concordance (KCC) value (also called ReHo value) was calculated to measure the similarity of the ranked time series of a given voxel to its nearest 26 neighbor voxels [22]. By calculating the KCC value of every voxel in the whole brain, an individual ReHo map was obtained for each subject. All individual voxel-wise ReHo values were computed and standardized into ReHo *z*-values by subtracting the mean ReHo obtained from the entire brain (i.e., global ReHo, male 0.1632 ± 0.0205, female 0.1578 ± 0.0113, *P* > 0.05) and then dividing by the standard deviation [25–27]. Spatial smoothing was then performed with a Gaussian filter of 8 mm full-width half-maximum (FWHM) kernel in order to manage the anatomical variability and to improve the signal-to-noise ratio [13]. Before the intergroup comparison, all ReHo maps were spatially normalized to the standard Talairach atlas template.

### 2.5. Intergroup Analysis

To explore the ReHo differences between the male and the female groups, a second-level, random-effect, two-sample two-tailed* t*-test was performed on the individual normalized ReHo maps in a voxel-by-voxel manner within the brain. Although age was not significantly different between groups, it was still included as a covariate to avoid any possible influence. The AFNI Monte Carlo simulation program AlphaSim was used to obtain a corrected significance level of *P* < 0.0001 and a minimum cluster size of 4 voxels (108 mm^3^) in the group difference maps. The anatomical localization and labeling of the functional data was determined by both Talairach coordinates and three radiologists.

### 2.6. RSNs Analysis

For RSNs analysis, the regions of interest (ROIs) of 12 RSNs were derived from our previous study [24]. Using ICA analysis, the preprocessed time series of BOLD after head motion correction, smoothing, and spatial normalization were concatenated along time to form a 4-dimensional (4D) dataset. GIFT-toolbox [28] was used to decompose the 4D BOLD data into 20 mutually independent components. These analyses identified 12 RSNs for BOLD data. There were the DMN, left attention networks (LAtN), right attention network (RAtN), primary visual network (PVN), secondary visual network (SVN), SMN, AN, executive network (EN), dorsal medial prefrontal network (DMPFC), ventral medial prefrontal network (VMPFC), salience network (SN), and medial temporal limbic network (MTLN). The ROIs' ReHo of each subject was extracted from the individual normalized ReHo maps, and the mean ReHo of each subject was calculated by the program of 3dROIstats in the AFNI software. The ReHo of 12 RSNs was statistically compared between males and females using independent samples* t*-test approach. The threshold was defined as the Bonferroni correction of *P* < 0.05 (i.e., *P* < 0.05/12 = 0.00417). Further, we also did an exploratory analysis (the threshold was set as *P* < 0.05), which reflects the exploratory nature.

## 3. Results

### 3.1. General Information

Fifty-six cases of males (mean age: 25.93 years, range 20–43 years) and fifty-six cases of females (mean age: 26.46 years, range 18–44 years) were finally included in the second-level analysis. Age was not significantly different between groups. All subjects in this study had less than 2 mm translation and 2° of rotation in any of the *x*-, *y*-, and *z*-axes. Therefore, no subjects were removed from the data analysis.

### 3.2. Cerebral ReHo Differences between Males and Females

**Figure 1:**
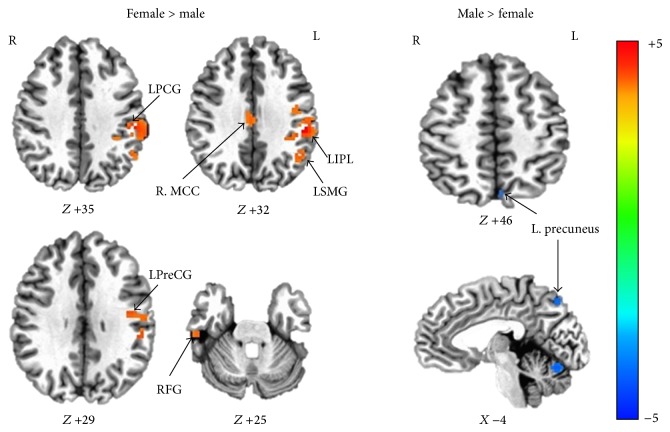
ReHo differences of cerebral regions between males and females (Monte Carlo simulation program AlphaSim, *P* < 0.0001, cluster size ≥ 4, and *α* < 0.05). Women showed greater ReHo than men in areas of the right middle cingulate gyrus (R. MCC), right fusiform gyrus (RFG), left inferior parietal lobule (LIPL), left precentral gyrus (LPreG), left supramarginal gyrus (LSMG), and left postcentral gyrus (LPG), while men showed greater ReHo in the left precuneus. L, left; R, right.

It was showed that there existed significant differences in the intergroup comparison between genders. Significantly higher ReHo in the left precuneus was found in the males, and significantly higher ReHo in the right middle cingulate gyrus, right fusiform gyrus, left inferior parietal lobule, left precentral gyrus, left supramarginal gyrus, and left postcentral gyrus was found in the females (Table 1 and Figure 1).

### 3.3. ReHo Differences of RSNs between Males and Females

**Figure 2:**
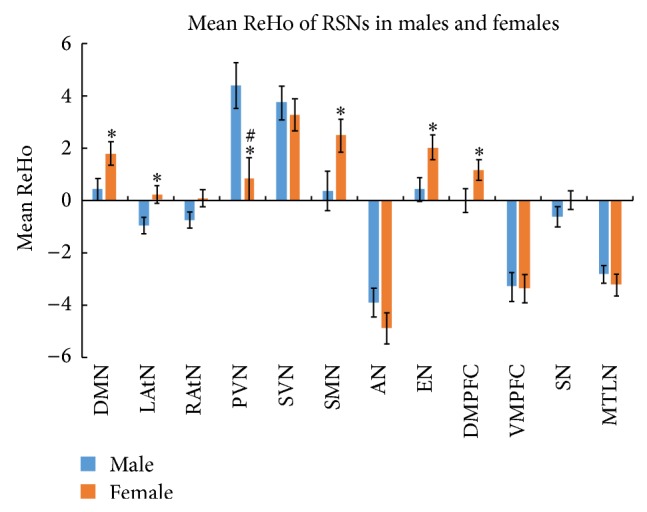
Mean ReHo of 12 RSNs between males and females. Significant differences were observed in the PVN (with Bonferroni correction, ^#^ *P* < 0.004). In the exploratory analysis, significant differences were observed in the DMN, LAtN, PVN, SMN, EN, and DMPFC as well as PVN (uncorrected, ^*^ *P* < 0.05). Error bar represented standard error.

The ReHo of PVN was found higher in males than females (*P* < 0.004). In the exploratory analysis, we further found higher ReHo of the DMN, LAtN, PVN, SMN, EN, and DMPFC in females compared to males (*P* < 0.05). No gender differences were found in the other 6 RSNs (Table 2 and Figure 2).

## 4. Discussion

This study applied ReHo analysis approach to investigate gender differences of resting-state in a large sample of adult healthy individuals. It was found that there existed gender difference in the PVN, LAtN, and some brain areas functionally related to gender differences in cognitive and behavior domains. These results suggest that males and females might have regional specific differences during the resting-state.

In the 12 RSNs, the higher ReHo of PVN was found in males than females, which showed significant gender difference between males and females after Bonferroni correction. PVN is associated with visual processing and SMN is related to motor function [23]. The higher ReHo of PVN in males might imply that males do better in visuospatial processing than females, which is consistent with previous anatomical and behavioral researches [11–13]. In the exploratory analysis, females had higher ReHo of DMN, LAtN, SMN, EN, and DMPFC than males. As previous studies reported, DMN, LAtN, EN, and DMPFC are involved in a wide range of cognitive processes and memory function [29, 30]. The results might suggest that women outperform men in some cognitive domains and emotion recognition processing, which is in line with previous researches [4, 17]. Several previous studies have reported the gender differences of functional brain networks during rest [30–32]. However, there were some consistency and some inconsistency as well. Our findings were consistent with the fact that there were no significant differences between sexes in the functional connectivity of the brain areas within the SN reported by Weissman-Fogel et al. [30], but they were inconsistent with our results of exploratory analysis that the EN and the DMN which they reported had no significant differences between genders. Another report [32] demonstrated that significant gender differences of resting-state activity were found in all networks. We suggested that the discrepancy might mainly result from the different methods they had used in their data analysis.

The specific brain areas in which gender difference of ReHo was found could be classified into two kinds. One kind was that female had higher ReHo and another kind was that male had higher ReHo. Six regions of greater ReHo were shown in females than males, including the right middle cingulate gyrus, right fusiform gyrus, left inferior parietal lobule, left precentral gyrus, left supramarginal gyrus, and left postcentral gyrus in the females. The inferior parietal lobule is a part of left attention network, which was identified from BOLD data using ICA analysis. It is concerned with multiple aspects of sensory processing and sensorimotor integration [33], especially in the perception of emotions in facial stimuli [34], and also, it is concerned with language and body image [35–38]. The middle cingulate cortex is involved in many different functions, including negative affect, pain, and cognitive control [39]. It has also reported that middle cingulate cortex receives widespread inputs, both directly and indirectly, from emotion-related brain regions [40] and may be a pivotal node of emotion and motor integration [41, 42]. Mann et al. [43] observed that women had relatively larger cingulate gray matter volume than men and showed different patterns of age-related volume decline between men and women. Although there are still some disputes on the functionalities of fusiform gyrus, a relative convergent point is related to various cognitive functions [44], such as face and body recognition [45–48], orthography and reading [49–51], word recognition [52], and processing of color information [53–56]. Therefore, the higher ReHo of the above areas in females is consistent with the behavioral sex difference that women generally excel in language [14, 15], facial emotion recognition [16], and emotional memory tasks [4, 17]. Furthermore, the different ReHo areas of the precentral gyrus between genders were located in its inferior part, which represents the primary motor area of hand and orofacial area [57]. The anterior supramarginal gyrus, which showed regional specific differences between genders in the present study, is also a component of left attention network in our results of ICA processing. It is involved in tool action observation [58]. Consistent with the previous studies [4, 14–17], these results might suggest that women excel better in hand and orofacial related tasks, something like fine motor skills, facial expression, and verbal fluency.

Meanwhile, we found that higher ReHo in the specific region of the left precuneus in men than in women. The precuneus is a part of PVN in our network-wise analysis ROIs. It is strongly interconnected with the parietooccipital visual and oculomotor-related cortices [59]. The precuneus is involved with visuospatial processing [60–62], episodic memory [63], reflections upon self [64, 65], and awareness and conscious information processing [66, 67]. These different processes may be selectively related to different subregions within the precuneus [61]; for example, the posterior subregion, a part of PVN, was related to visual area [68]. In this study, the posterior subregion of left precuneus showed higher regional homogeneity in men, which was consistent with the previous report [61]. In addition, we also found the higher ReHo of the PVN in males than females. Therefore, it might reflect that there existed difference in visuospatial processing between men and women [11, 12] and provided further evidence to a converging point suggested by previous behavioral study; that is, men generally perform better in visuospatial processing than women [11–13].

A gender difference in cerebral regional homogeneity of adult healthy volunteers was also reported by Wang et al. [13]. There were some consistency and inconsistency between their results and ours. The consistency was that both Wang and we found gender difference in resting-state function of healthy volunteers and women mainly exhibited higher ReHo in their left hemispheres. In this study, we adopted the independent samples and repeated the results of left hemispheres preponderance of ReHo in females, which strengthened the conclusion of the higher ReHo in females' left hemispheres concluded by Wang et al. [13]. However, the specific brain regions showing gender differences were not exactly the same. We thought the discrepancy mainly resulted from the difference in methodology. In Wang's study, a support vector machine-based MVPA approach was employed to identify the complex patterns of sex differences in brain structure and resting-state function, but in our study, the ReHo maps were compared between the males and the females using* t*-test. Different data analysis methods can demonstrate different results [69, 70], and the threshold selection can also influence the results [71]. Thus, the result difference between this study and Wang's study might indicate different aspects of resting-state brain function via different analysis methods. In addition, age range may have an effect on resting-state homogeneity [21, 72, 73]. Therefore, we would use different analysis methods and pay more attention to the effect of age in future studies in order to make the results more comprehensive.

Potential limitations of this study should be noted. First, in our study, age range of subjects is a little broader for men than women. As previous researchers reported [21, 72, 73], age range is critical as it may have an effect on resting-state homogeneity. Although age was not significantly different between our groups and we had included age as a covariate to avoid any possible influence, it would be better to increase the sample size and minimize the age range in the future investigations. Second, the present study had not correlated the ReHo value with the behavioral data for no such data were collected in the experiment. In the future study, this factor should be taken into account. Third, although it is a general practice that global signal of ReHo was removed when doing group level analysis, Zuo et al. [25] reported that removing global effect would reduce reliability of ReHo. Therefore, we should improve our data processing methods in the future. Fourth, the results of DMN, LAtN, SMN, EN, and DMPFC in the present study are based on the exploratory analysis (i.e., it did not reach the threshold after Bonferroni correction although some of them are close). Therefore, these results should be replicated in the future.

In summary, the present study found gender differences in regional homogeneity of adult healthy volunteers within some of RSNs and cerebral cortices and indicated that men and women might have regional specific differences during the resting-state. Many of the specific regions showed in voxel-wise analysis belong to the RSNs that showed gender differences in the present study. These regional specific regions are mostly related to the functions of behavior and cognition. The findings are consistent with the gender differences in behavioral and cognitive domains and might improve our understanding of the gender differences from the perspective of brain function.

## Figures and Tables

**Table 1:** Cerebral gender differences in ReHo between males and females.

Regions	Brodmann area	Side	Coordinate (Talairach)			Voxels	*z*-value
			Peak *x*	Peak *y*	Peak *z*		
Female > male							
Inferior parietal lobule	BA40	L	−46.5	−31.5	32.5	43	4.83
		L	−40.5	−34.5	32.5	8	4.26
Precentral gyrus	BA6	L	−37.5	−10.5	29.5	17	4.32
Supramarginal gyrus	BA40	L	−46.5	−52.5	32.5	13	4.2
Middle cingulate gyrus	BA24	R	1.5	−19.5	32.5	10	4.17
Fusiform gyrus	BA20	R	52.5	−22.5	−24.5	4	4.09
Postcentral gyrus	BA2	L	−46.5	−25.5	35.5	4	4.43

Male > female							
Precuneus	BA7	L	−4.5	−67.5	47.5	4	−4.09

Note: BA, Brodmann area; L, left; R, right. The threshold was set to *P* < 0.0001, *α* < 0.05, and cluster ≥ 4 (Monte Carlo simulation program AlphaSim).

**Table 2:** Comparison of ReHo in RSNs between males and females.

	DMN	LAtN	RAtN	PVN	SVN	SMN	AN	EN	DMPFC	VMPFC	SN	MTLN
Male	0.411	−0.982	−0.759	4.390	3.737	0.356	−3.922	0.412	−0.003	−3.314	−0.637	−2.838
Female	1.780	0.226	0.069	0.810	3.257	2.475	−4.904	2.021	1.157	−3.387	−0.007	−3.245
*t*	2.258	2.741	1.803	−2.974	−0.538	2.186	−1.215	2.47	1.988	−0.095	1.243	−0.757
*P*	0.026^*^	0.007^∗#^	0.074	0.004^*^	0.592	0.031^*^	0.227	0.015^*^	0.049^*^	0.925	0.217	0.451

Note: DMN, default mode network; LAtN, left attention networks; RAtN, right attention network; PVN, primary visual network; SVN, secondary visual network; SMN, sensorimotor network; AN, auditory network; EN, executive network; DMPFC, dorsal medial prefrontal network; VMPFC, ventral medial prefrontal network; SN, salience network; MTLN, medial temporal limbic network. ^#^Independent samples *t*-test, significance threshold *P* < 0.004 (with Bonferroni correction). ^*^Independent samples *t*-test, significance threshold *P* < 0.05 (uncorrected).
